# Are Induced Pluripotent Stem Cells a Step towards Modeling Pediatric Leukemias?

**DOI:** 10.3390/cells11030476

**Published:** 2022-01-29

**Authors:** Salvatore Nicola Bertuccio, Davide Leardini, Daria Messelodi, Laura Anselmi, Francesca Manente, Federico Ragni, Salvatore Serravalle, Riccardo Masetti, Andrea Pession

**Affiliations:** 1 Department of Medical and Surgical Sciences (DIMEC), University of Bologna, 40138 Bologna, Italy; salvatore.bertuccio2@unibo.it (S.N.B.); francesca.manente2@studio.unibo.it (F.M.); federico.ragni2@studio.unibo.it (F.R.); riccardo.masetti@gmail.com (R.M.); 2 Specialty School of Pediatrics, University of Bologna, 40138 Bologna, Italy; davide.leardini3@gmail.com; 3 Pediatric Oncology and Hematology Unit “Lalla Seràgnoli,” Pediatric Unit, IRCCS, Azienda Ospedaliero-Universitaria di Bologna, 40138 Bologna, Italy; salvatoreserravalle@libero.it; 4 Division of Pediatrics, IRCCS, Azienda Ospedaliero-Universitaria di Bologna, 40138 Bologna, Italy; andrea.pession@unibo.it

**Keywords:** pediatric leukemia, in vitro modeling, iPSCs, genome editing, new target therapies

## Abstract

Despite enormous improvements in pre-clinical and clinical research, acute leukemia still represents an open challenge for pediatric hematologists; both for a significant relapse rate and for long term therapy-related sequelae. In this context, the use of an innovative technology, such as induced pluripotent stem cells (iPSCs), allows to finely reproduce the primary features of the malignancy and can be exploited as a model to study the onset and development of leukemia in vitro. The aim of this review is to explore the recent literature describing iPSCs as a key tool to study different types of hematological malignancies, comprising acute myeloid leukemia, non-down syndrome acute megakaryoblastic leukemia, B cell acute lymphoblastic leukemia, and juvenile myelomonocytic leukemia. This model demonstrates a positive impact on pediatric hematological diseases, especially in those affecting infants whose onsets is found in fetal hematopoiesis. This evidence highlights the importance of achieving an in vitro representation of the human embryonic hematopoietic development and timing-specific modifications, either genetic or epigenetic. Moreover, further insights into clonal evolution studies shed light in the way of a new precision medicine era, where patient-oriented decisions and therapies could further improve the outcome of pediatric cases. Nonetheless, we will also discuss here the difficulties and limitations of this model.

## 1. Introduction

Acute leukemia (AL) represents the most frequent cause of childhood cancer-related mortality worldwide, with an estimated incidence of 10 to 50 cases per 100,000 every year and a cumulative risk of 1 in 2000 up to the age of 15 [1]. Acute lymphoblastic leukemia (ALL) is most frequent subtype, representing about 80% of all cases of leukemias [2], while acute myeloid leukemia (AML) represent a rarer subtype [3]. Another form of myeloid malignancies is juvenile myelomonocytic leukemia (JMML), which represents a clonal disorder of early childhood characterized by hyperactivation of RAS signaling pathway [4]. Currently, cure rates for ALL are around 90%, representing an often-cited paradigm of success in modern clinical and translational research-based medicine [5]. In addition, treatment outcomes for pediatric patients with AML lagged behind outcomes reported for ALL, even if they significantly improved [3] over time, reporting a cure rate slightly higher than 70%. This “history of success” found its basis on years of translational research focusing on a deep characterization of the disease and treatment according to risk stratifications. Deep genetic characterization has been widely performed on pediatric ALL, AML, and JMML opening new research and treatment scenarios [6]. Clinical studies have been performed to assess the role of different genetic features on clinical outcomes [7]. However, other in vitro and in vivo study platforms are needed to directly address the effect of these features.

In vivo mouse models of pediatric acute leukemia have been widely established, providing important clues in biological mechanisms. The classic method to model leukemia in mice is to induce the expression of mutations or fusion genes into hematopoietic stem progenitor cells (HSPC) derived from mouse adult bone marrow via a retroviral vector [8]. However, this method carries several limitations. First of all, the expression levels of the fusion genes do not always reproduce the in vivo levels and this creates difficulties in understanding their biological role, especially for highly expressed genes. Another very important limitation is related to the specificity, in terms of origin and functions of hematopoietic cells. Indeed, many pediatric leukemias, in particular in infant varieties, have been shown to develop in utero, reflecting their fetal origin. It is thus believed that driving mutations most likely need to be active in that precise moment to result in leukemia onset [9]. Therefore, hematopoietic progenitors derived from adult mouse bone marrow are not able to reproduce with high fidelity the phenotype of pediatric leukemia disease [10].

Accordingly, many of the studies focused on KMT2A-rearranged leukemia, the most translocated genes in infant leukemia, demonstrated that expression of KMT2A-MLLT3 in fetal liver-derived HSPC gives rise to a leukemic phenotype often expressing lymphoid surface markers. On the other hand, transplantation of KMT2A-MLLT3 adult BM-derived cells leads to the typical AML-M5 phenotype [11], corresponding to the phenotype of patients and suggesting that activation at a particular developmental stage significantly influences disease biology. Currently, there is a lack in animal models reproducing KMT2A fusion-driven pediatric AML. Although the generation of mouse models that can mimic the prenatal origin of the disease could be an important contribution to the repertoire of research tools for pediatric leukemia, animal models still carry other important limitations in recapitulating the human leukemic phenotype, mainly due the presence of an embryonic lethality, a non-hematopoietic expression or the upraise of a lymphoma. Another crucial concern about the use of animal models is the requirement of dedicated facilities, involving economic issues and manpower. Certainly, animal models have contributed tremendously to a better understanding of disease mechanisms. However, these limitations, alongside difficulties in terms of an accurate recapitulation of human diseases and drug testing poses important limits [12].

On the other hand, the in vitro disease modeling has some limitations related to the difficulties in keeping primary tumoral cells in culture and the use of tumoral cell lines that do not represent at best the pathological condition because of the numerous genetic alterations. As an example, it was observed in different studies that JMML progenitors cultured in vivo give rise to an excessive formation of monocyte-macrophage colonies. In fact, maintaining immature JMML progenitor cells in culture poses a big challenge, as they tend to differentiate and undergo rapid senescence. Indeed, it is possible to maintain undifferentiated JMML cells for approximately 2 weeks in medium supplemented with different types of cytokines [13]. Moreover, AML primary blasts are difficult to maintain in culture as well, due to rapid differentiation and undergoing apoptosis in ex vivo cultures, and they often need the bone marrow support or addition of small molecules such as Aryl hydrocarbon suppressor [14].

Given these difficulties and paucity of primary samples, new research methods are needed to investigate and deeply characterize childhood leukemias in order to better understand the underlying biology and to discover new therapeutic targets [15].

## 2. Induced Pluripotent Stem Cells in Pediatric AL Research

In this context, the use of induced pluripotent stem cells (iPSCs) has become particularly attractive. iPSCs are pluripotent stem cells derived from different types of adult somatic cells, which have been genetically reprogrammed to an embryonic stem (ES) cell-like state through the reactivation of the expression of genes important for maintaining the defining properties of ES cells. This condition is reached by means of cell transfection with vectors encoding for these factors: Oct4, Sox2, Myc, and Klf4. iPSCs are able to proliferate, maintaining pluripotency, and to differentiate in the three embryonic germline layers in vitro. In recent years, iPSCs have been considered an emerging approach to model human diseases and it has been thought to be applied in the field of personalized medicine. iPSCs represent an ideal tool in disease modeling for several reasons. First, iPSCs can proliferate indefinitely proving a long-lasting cell line. Moreover, they can be differentiated in different cell type allowing to test the effect of mutations on different tissues. Lastly, iPSCs can provide a biological tool to test treatments on patient-specific cells [12,16].

In a theoretical way, all the human somatic cell types can be reprogrammed to generate iPSCs colonies, but in practicality only some types of cells have been reprogrammed successfully, including fibroblasts, peripheral blood mononuclear cells, and CD34+ hematopoietic progenitors [17]. In the field of onco-hematology, several research groups tried to reprogram pediatric leukemia cells from patients and healthy donor, generating iPSCs, carrying the disease causative genetic alterations.

For the subsequent in vitro differentiation into mature hematopoietic cells to fully reproduce features of leukemia stem cells (LSCs) and leukemic blasts, many differentiation protocols have been developed, enabling the creation of a novel and more efficient platform for leukemia research and for patient-specific immunological treatment development. Yang Li. et al. [18] succeeded in the generation of iPSCs from different types of pediatric acute leukemia patients, transducing the cells with the Sendai virus encoding for the four reprogramming factors, and all cell lines maintained features of pluripotency and the capacity of in vitro differentiation. In particular, they reprogrammed three types of leukemia cells: T-ALL, AML M7, and AML M5, demonstrating that [18] some types of leukemic cells are refractory to reprogramming [18].

The following chapters will highlight and recapitulate the main achievements in terms of disease iPSC modeling and the challenges that are still ongoing considering the different types of leukemia involved.

## 3. Modeling of Pediatric Acute Myeloid Leukemia (AML)

As briefly mentioned, AML is still an open challenge for pediatric hematologists, as its overall good survival rate is accompanied by recurrence of the disease in about 25% of patients [19]. Despite a better understanding of the biology of AML, modest progress in treatment outcome has been made compared to ALL. The main reasons for these differences are the heterogeneity of the disease, a paucity of available targeted therapies, and the lower effectiveness of immunotherapy. In addition, the chemotherapeutic intensity has been raised to the highest tolerated degree and it is highly unlikely that further intensification would increase survival rates. Data from large AML cooperative studies are providing deep genomic analyses of the leukemic stem cell, revealing new insights into the biology on the AML and new therapeutic targets. Furthermore, new therapies are being introduced in recent years, such as BCL-2 inhibitors, CD33- and CD123-directed chimeric antigen receptor T-cell therapy, and CD123-directed antibody therapy. Pediatric AML can be associated with different fusion oncogenes and this association strongly correlates with the patient age [20]. The most common fusion genes include KMT2A (MLL), CBFA2T3, MNX1, RUNX1, CBFB, RARA, or NUP98 translocations. AML cells, in comparison to other type of leukemia, are sometimes resistant to reprogramming [21]. Indeed, Jong-Hee Lee et al. [21] attempted to reprogram AML blasts harboring the most common fusion genes. However, most AML blasts are refractory to the reprogramming process except for the MLL-AF9 fusion gene with a great variability between patients, suggesting that expression of MLL fusion is not sufficient to improve the efficiency of the reprogramming process. Given the susceptibility of MLL blast to the reprogramming process, the iPSC model offers the possibility to study different types of clones separately. Indeed, most cases of AML are composed of a mixture of different subclones which display variable genetic features. The presence of subclonal diversity has prominent implications in disease progression, drug response, relapse, and development of targeted therapies. As iPSCs are derived from single cells, giving rise to different colonies, clones can be separated and finely characterized, thus allowing cancer studies to focus on such diversification. Chao et al. [22] investigated multiple aspects regarding AML by questioning an AML-iPSC model. They successfully reprogrammed human AML cells carrying MLL rearrangements into iPSCs, which retained the original cytogenetic aberration. Nonetheless, these AML-iPSCs, whenever differentiated into non-hematopoietic lineages, exhibited normal cellular morphology and functions, and their leukemic potential was evicted only after hematopoietic differentiation. This was demonstrated by the aberrant myeloid phenotype and colony forming potential in vitro, and their ability to engraft and give rise to aggressive myeloid leukemia in vivo. Interestingly, when reprogrammed into iPSCs, AML blasts reset their DNA methylation and epigenetic pattern, which is subsequently reacquired upon hematopoietic differentiation. Moreover, they demonstrated that this phenotype is not linked to any residual epigenetic memory, but rather an MLL-dependent signature which is activated only after hematopoietic differentiation occurs.

Other research groups tried to model other subgroups of pediatric AML. The first successful generation of an iPSC line from HL-60 human immortalized acute myeloid leukemia (AML) cell line was performed by the research group of Amanda E. Yamasaki, through the use of Sendai virus. HL-60-iPSCs demonstrated to maintain the genetic aberrations of the parental HL-60 leukemic line, the pluripotency and the capacity to generate cells expressing genes for all the three embryonic germ layers was tested, while a partial block in the differentiation into hematopoietic mature cells expressing CD16 marker was reported [23].

AML1-ETO (t(8;21)) is a common chromosomal translocation in AML that generates an oncofusion protein, most of the time associated with a good prognosis. AML1-ETO is well characterized in mouse models, AML patient blasts, and cell lines (such as Kamusi-1 cell line). The group of Esther Tijchon also managed to generate iPSCs allowing the study of the expression of the fusion protein during in vitro differentiation thus having a useful model to analyze the deregulated transcriptome and epigenome. They proved that AML1-ETO specifically affects the development of the granulocyte lineage, reducing the numbers of CD15+CD16+ granulocytes and increasing the CD34+CD45+ progenitors, without affecting monocyte differentiation [24].

Moreover, iPSC-based models represent a key tool to be exploited in clonal evolution studies. A challenging question that arose in human cancers characterization is how to describe the evolutionary process through which somatic mutations accumulate sequentially and drive clonal selection, thus inducing the malignant phenotype. The identification of disease specific stages and transitions could help to highlight the sets of genes sufficient to promote cell transformation, but also to illuminate targeted therapeutic alternatives at an early disease stage. As a matter of fact, leukemic blasts are extremely difficult to manipulate for in vitro pharmacologic studies, as patients’ samples are often limited in number and rapidly undergo apoptosis when placed into culture. On the contrary, iPSCs derived from patients could contribute to investigation of targeted therapies based on specific mutations or subclones.

Wang et al. [25] contributed to dissect AML clonal evolution thanks to the combination of iPSC and CRISPR-Cas9 technology, an extensive work in part already published before, dissecting stage-specific paths from myeloid transformation to malignancy [26]. Based on population genetic studies, they previously selected a panel of recurrent mutations found in secondary AML (i.e., arising from preexisting MDS) and introduced them stepwise in karyotypically normal iPSCs derived from a MDS patient. Sequentially, ASXL-1 C-terminal truncation, SRSF2 P95L, and NRAS G12D mutations were introduced, all in a heterozygous state, by means of CRISPR-mediated gene editing. A fourth mutation, FLT3-ITD, was included afterwards. As FLT3 and RAS mutations are known to be mutually exclusive in AML, they expected redundancy between these mutations. At each stage after hematopoietic differentiation, these iPSCs finely recapitulate AML transcriptional features; through grouped analysis of ATAC-seq peaks, early events during AML developments were highlighted. As a matter of fact, these initial genes were all linked to inflammatory responses and immune signaling, and two preclinical compounds that inhibit inflammatory signaling (a dual inhibitor of IL-1 and IRAK4, and an inhibitor of a ubiquitin-conjugating enzyme required for innate immune signaling) displayed efficient activity in disrupting colony formation at all mutational stages in a dose-dependent manner, excluding the wild-type parental iPSCs. Accordingly, this study revealed the importance of iPSCs to generate specific lines with progressive malignant mutations that are coherent with the clinical features expressed by human premalignant and malignant myeloid pathologies. Moreover, they also studied the in vivo engraftment in mice of these iPSCs, which previously gave good results only with AML patients-derived iPSCs. Interestingly, they were able to recapitulate the in vitro and in vivo phenotypes displayed by disease free, clonal hematopoiesis, MDS, and sAML.

As previously seen, AML-iPSCs enable the possibility to characterize and specifically target distinct AML subclones which might display distinct mutational features and not respond to other conventional treatments, and eventually represent those clones responsible for resistance episodes or future relapse.

## 4. Non-Down-Syndrome Acute Megakaryoblastic Leukemia

Non-Down syndrome acute megakaryoblastic leukemia (non-DS-AMKL) is a heterogeneous form of acute myeloid leukemia (AML), defined by the presence of leukemic megakaryoblastic cells, representing about the 4–15% of pediatric AML [27,28]. Despite the low incidence, there is a strong clinical interest to characterize the leukemogenic mechanism, as well as to identify new therapeutic targets for this AML subgroup. Indeed, while children with Down syndrome-AMKL show a favorable outcome [29], non-DS-AMKL has been historically associated with a poor prognosis [7]. About half of non-DS-AMKL harbors recurrent fusion genes. The most common are ETO2-GLIS2 [30], NUP98-KDM5A, and KMT2A-MLLT3 (MLL-AF9) [28].

Independently of the driver fusion it has been demonstrated that non-DS-AMKL has fetal origin. Indeed, expression of ETO2-GLIS2 in hematopoietic progenitors derived from mouse fetal liver recapitulated AMKL features phenotype [31]. In this context, iPSCs provide a unique tool based on human cells to explore predisposition to leukemia transformation of fetal hematopoiesis. Recently, a healthy iPSC line was engineered to express the gene fusion ETO2-GLIS2 via zinc finger nucleases under the control of pan hematopoietic promoter CD43. Differentiation of this iPSC line into hematopoietic progenitors reproduced the principal features of AMKL patients, including the increasing of aberrant megakaryocytes, self-renewal, and expression pathway similar to AMKL pediatric patients. However, hematopoietic progenitors-iPSC derived expressing ETO2-GLIS2 failed to engraft in NSG mice. Hypotheses have addressed a variety of scenarios: the expression level of fusion gene is not sufficient to completely transform megakaryocytes into AMKL or CD43 promoter could select a primitive erythroid population CD43+ that is the first primitive population emerged from iPSC differentiation protocol [32]. Although further investigations need to clarify this important issue, hematopoietic progenitors derived from iPSCs and expressed fusion oncogenes are of course suitable to test efficacy and security of new target drugs.

## 5. B-Cell Precursor Acute Lymphoblastic Leukemia

B-cell precursor ALL (BCP-ALL) is the most frequent ALL subtype in children. It is an heterogeneous disease driven by different types of initiating genetic alterations: chromosomal aneuploidy, rearrangements, and point mutations [2] occurring in different genes, responsible for cytokine-receptor or kinase signaling, such as ABL1, ABL2, PDGFRB, CSF1R, JAK2, RUNX1, EPOR, and CRLF2 [33]. iPSCs modeling of ALL represented a harder goal compared to AML. Munoz-Lopez et al. first reported attempts to establish an iPSC-based ALL model by OKSM(L)-expressing mono/polycistronic-, retroviral/lentiviral/episomal-, and Sendai virus vector-based reprogramming strategies without success. Interestingly, they succeeded in reprogramming MLL-AF4 expressing B progenitors suggesting that B cell origin and leukemic fusion gene were not reprogramming barriers [34]. These difficulties may be also ascribable to the complex karyotype often present in ALL blasts resulting in high cell instability. Li et al. first succeeded in reprogramming primary T-ALL blasts in iPSCs even with some bias in the study [16].

In addition, genome-engineered iPSCs ALL models have been established with the main focus on unraveling the origin of leukemia. There is evidence that initiating mutations are frequently acquired in utero [35], as it happens for the ETV6-RUNX1 (TEL-AML1) fusion gene that accounts for 25% of precursor BCP-ALL in children. This alteration represents a frequent initiating event, even if only 1% of children with the ETV6-RUNX1 mutation develop the second-hit mutations required to transform to overt ALL, suggesting its weakly penetrant first-hit oncogene nature [36]. In this context, iPSCs carrying the ETV6-RUNX1 fusion gene have been exploited as a suitable tool to examine the earliest stages of human fetal B lymphopoiesis and to understand how ETV6-RUNX1 initiates the pre-leukemia condition in utero. ETV6-RUNX1 expression was reported to specifically affect the transition from fetal IL-7R+ progenitor compartment to committed proB cell causing a partial block in B lineage commitment, and the generation of proB cells with aberrant myeloid gene expression signatures and potential [36].

Fortschegger et al. [37] established an iPSCs model focusing on the RUNX1-JAK2 fusion gene studying its consequences on hematopoietic development though the insertion of the RUNX1-JAK2 fusion into one endogenous RUNX1 allele employing in trans paired nicking genome editing. They observed a decrease in the hematopoietic progenitor generation that, nonetheless, did not result in the lack of myeloid lineage cell differentiation. Moreover, RNA-seq performed RUNX1-JAK2-expressing hematopoietic cells showed the constitutive activation of JAK-STAT signaling and the upregulation of MYC targets, confirming the role and interaction between these pathways in the onset of the leukemic process. However, increased clonogenicity or enhanced hematopoietic differentiation upon RUNX1-JAK2 expression were not observed, hypothesizing that hematopoietic progenitors derived from iPSC were not susceptible to oncogenic transformation [37].

## 6. Juvenile Myelomonocytic Leukemia (JMML)

Juvenile Myelomonocytic Leukemia (JMML) is a myeloproliferative neoplasm that affects mainly infants and young children, driven by Ras pathway mutations, hyperactivation of Ras/Mitogen-activated protein kinase pathway and PI3K/mTOR signaling leading to excessive formation of leukemic cells in the myelomonocytic and red cell lineage. The constitutive activation of the Ras/MAPK pathway is caused by some mutually exclusive different type of somatic or germline loss-of-function or gain-of-function mutations, such as by PTPN11 (protein-tyrosine phosphatase, non-receptor-type 11; encoding SHP2), Kirsten rat sarcoma virus (KRAS), neuroblastoma RAS viral oncogene homolog (NRAS), neurofibromatosis type 1 (NF1) or Casitas b-lineage lymphoma (CBL) in the leukemic cells. Furthermore, children with Noonan syndrome (NS; OMIM163950), a genetic disorder with increased RAS/MAPK signaling, are predisposed to developing JMML. Fifty percent of NS patients with JMML cases carry gain-of-function PTPN11 mutations [38] (pp. 620–631). JMML is characterized by splenomegaly, thrombocytopenia, peripheral monocytosis, elevated hemoglobin F, and hypersensitivity to granulocyte macrophage colony stimulating factor (GM-CSF). The only effective therapy for JMML is the allogeneic hematopoietic stem cell transplantation (HSCT), even if there is still the risk of post-HSCT relapse and end-organ infiltration suggesting the need for new target therapies.

However, JMML remains a difficult disease to study given its rare incidence and paucity of primary samples. In this context the iPSC model offers a unique opportunity to reproduce the disease in vitro, as well as to perform drug testing. Moreover, despite the low reprogramming efficiency of pediatric AML samples with common aberrations, JMML carrying typical associated mutations, in particular PTPN11, are prone to reprogramming process.

Two independent studies reported reprogrammed peripheral blood from de novo JMML patients with PTPN11 [39,40] mutation and CBL mutation associated with chromosome 11q isodisomy [40]. The generated iPSC retains PTPN11 mutation and loss of heterozygosity of CBL, as well as pluripotency markers and morphology features. The PTPN11 and CBL mutant hematopoietic progenitors generated an increased proportion of myeloid cells compared with controls. Moreover, the clonogenic assay displayed an increment of myeloid colonies in presence of low/absence GM-CSF cytokine indicating constitutive activation of this cytokine signaling pathway [39,40]. Indeed, they observed constitutive activation of Ras/MAPK signaling in PTPN11 and CBL mutant myeloid cells. In addition, CBL myeloid cells displayed more elevated levels of JAK2 and STAT5 activation than PTPN11, while similar hyperactivation of PI3K/Akt/mTOR signaling was observed. Based on these observations, the drug screening test demonstrated sensitivities of JMML iPSCs to specific kinase inhibitors with some variations between the two subgroups. Indeed, PTPN11 had preferential sensitivity to MEK inhibition [39] while CBL cells had preferential sensitivity to JAK inhibition treatment [40]. Furthermore, at the molecular level PTPN11-JMML is associated with up-regulation of CDKN1A and MYC in myeloid cells, as well as up-regulation of miR-223 and -15a. The up-regulation of miR-223 mediated by PTNP11 mutation is sufficient to perturb myelopoiesis and downregulation of target genes such as FOXO3, SPTB, NPM1, WHSC1K1, DICER1, MTL5, RAB12, SMAP1, and SHROOM4 [41].

More recently, a proteomic analysis revealed a set of proteins deregulated in PTPN11 JMML, such as ITGß2 and S100A4. Gene ontology analysis revealed leukocyte migration as an altered linked pathway. This observation was functionally demonstrated and associated with TP53 and NF-κb as potential regulators [42]. The drug screening with NF-κb, TP53, and MYC inhibitors was performed. Nutlin inhibits the interaction between MDM2 and TP53 leading to the stabilization of TP53, JQ1 is a BET bromodomain inhibitor, which reduces transcription by disruption of chromatin-dependent signaling with MYC as a primary target and CBL0137 inhibits NF-κb, activates TP53, and has been reported to regulate MYC expression [43,44,45,46,47]. Except Nutlin, all of the drug combinations had an effect on colony formation in relation to nontreated controls. In particular, CBL0137 had a significant differential effect on the ability of PTPN11-JMML cells to form colonies [40,42].

## 7. Conclusions and Future Perspectives

**Figure 1:**
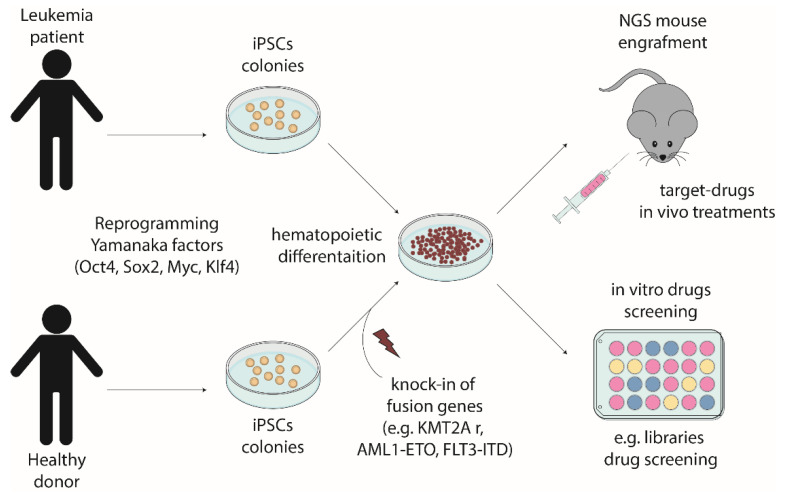
Modeling pediatric leukemia exploiting iPSC technology.

The generation of iPSCs model for childhood leukemia represent a huge step forward in the research on hematological malignancies (Figure 1). The iPSC-derived leukemia cells can reproduce the primary features of the disease and can be used to set a research model to analyze the onset and development of leukemia in vitro. The studies confirmed the iPSCs derived from leukemia expressed the same pluripotency genes as the embryonic stem cells, and their differentiation could be particularly useful when appropriate patient samples are not available and animal models cannot generalize human phenotypes [48]. Nonetheless, it is important to highlight the difficulties behind iPSCs. Indeed, accurate repeatable standard differentiation protocols need to be set up for each stage of the hematopoietic way. Furthermore, large scale data on multiple sets of patients-derived iPSCs have to be validated, in particular in the idea of being recognized as a trustful preclinical model.

From these premises, an intriguing perspective should include the creation of biobanks of iPSCs for all types of leukemia, recapitulating all the different fusion genes and genetic aberrations which can be found, to finely reproduce the phenotype of these diseases. This model would give the possibility to precisely dissect the biology of the disease and to evaluate the sensitivity to different mutation-specific therapies in children with different kinds of leukemia, as performed by the group of Tasian et al. [40]. Moreover, the creation of an iPSCs model offers the possibility to achieve a perfect in vitro representation of the human embryonic hematopoietic development is a revolutionary tool in the hands of scientists. Thanks to this model, it would be possible to dissect the whole leukemogenic process, to uncover the importance of the different ways of hematopoietic differentiation, from primitive to definitive, thus highlighting the fundamental role of timing-specific modifications, either genetic or epigenetic, in shaping the onset of a disease. Accordingly, this lays the groundwork for a more precise personalized therapy in pediatric leukemia to further consolidate the “history of success” and to develop new treatments for non-responding patients. Other than disease-focused interventions, in future iPSCs may serve as patient-focused decision making tools. Patient’s derived iPSCs cells may be set up to test the efficacy of different compounds either for antileukemic treatments or for supportive care treatments to further personalize the cure [12]. Progress in clinical medicine has always been made thanks to solid basic research, which enables the understanding of molecular processes and mechanisms underlying a specific pathological condition. Nonetheless, there is a great deal that needs to be still uncovered, and innovative tools, such as iPSCs, may represent the key to light our way. In this regard, the use of iPSC-based disease modeling in hematology is one out of hundreds of possible fields of application, which include solid tumors as well, with innovative 3-D organoids studies focused, for example, on gastrointestinal cancer [49].

## Data Availability

Not applicable.
